# Repositioning Azelnidipine as a Dual Inhibitor Targeting CD47/SIRPα and TIGIT/PVR Pathways for Cancer Immuno-Therapy

**DOI:** 10.3390/biom11050706

**Published:** 2021-05-10

**Authors:** Xiuman Zhou, Ling Jiao, Yuzhen Qian, Qingyu Dong, Yixuan Sun, Wei V. Zheng, Wenshan Zhao, Wenjie Zhai, Lu Qiu, Yahong Wu, Hongfei Wang, Yanfeng Gao, Junhui Chen

**Affiliations:** 1 Intervention and Cell Therapy Center, Peking University Shenzhen Hospital, Shenzhen Peking University-The Hong Kong University of Science and Technology Medical Center, Shenzhen 518035, China; H027@pkuszh.com (X.Z.); zhengw2013@yeah.net (W.V.Z.); 2 School of Life Sciences, Zhengzhou University, Zhengzhou 450001, China; jisunny@gs.zzu.edu.cn (L.J.); qyz_2021@gs.zzu.edu.cn (Y.Q.); 202022162012917@gs.zzu.edu.cn (Q.D.); sunyixuan@gs.zzu.edu.cn (Y.S.); zhaowsh07@zzu.edu.cn (W.Z.); wjzhai@zzu.edu.cn (W.Z.); qiulu@zzu.edu.cn (L.Q.); yahongwu@zzu.edu.cn (Y.W.); 3 School of Pharmaceutical Sciences (Shenzhen), Sun Yat-sen University, Shenzhen 518107, China

**Keywords:** CD47/SIRPα, TIGIT/PVR, drug-repositioning, small molecule inhibitor, azelnidipine, cancer immunotherapy

## Abstract

Strategies boosting both innate and adaptive immunity have great application prospects in cancer immunotherapy. Antibodies dual blocking the innate checkpoint CD47 and adaptive checkpoint PD-L1 or TIGIT could achieve durable anti-tumor effects. However, a small molecule dual blockade of CD47/SIRPα and TIGIT/PVR pathways has not been investigated. Here, an elevated expression of CD47 and PVR was observed in tumor tissues and cell lines analyzed with the GEO datasets and by flow cytometry, respectively. Compounds approved by the FDA were screened with the software MOE by docking to the potential binding pockets of SIRPα and PVR identified with the corresponding structural analysis. The candidate compounds were screened by blocking and MST binding assays. Azelnidipine was found to dual block CD47/SIRPα and TIGIT/PVR pathways by co-targeting SIRPα and PVR. In vitro, azelnidipine could enhance the macrophage phagocytosis when co-cultured with tumor cells. In vivo, azelnidipine alone or combined with irradiation could significantly inhibit the growth of MC38 tumors. Azelnidipine also significantly inhibits the growth of CT26 tumors, by enhancing the infiltration and function of CD8^+^ T cell in tumor and systematic immune response in the tumor-draining lymph node and spleen in a CD8^+^ T cell dependent manner. Our research suggests that the anti-hypertensive drug azelnidipine could be repositioned for cancer immunotherapy.

## 1. Introduction

Cancer immunotherapy represented by the blockade of immune checkpoint PD-1/PD-L1 has gained substantial progress. The anti-PD-1/PD-L1 resistance resulting from the upregulation of immune checkpoints suggests the importance and urgency of developing new targets [1]. A combinational blockade of different targets with non-redundant functions may achieve better clinical benefits. A novel immune checkpoint T cell immunoglobulin and immunoreceptor tyrosine-based inhibitory motif (TIGIT), which always co-expresses with PD-1 on NK and T cells and symbolizes a more exhausted status, plays pivotal roles in the adaptive anti-tumor immunity by ligation with its major ligand poliovirus receptor (PVR) [2,3]. PVR was initially identified as the receptor for the human poliovirus, or as an adhesion-related molecule that mediates tumor invasion. Increasing attention has been paid to PVR in cancer immunotherapy since the verification of its role as the shared ligand for the immune checkpoint TIGIT and CD96 [4,5,6,7]. The elevated expression of PVR facilitates the immune escape of tumor cells including melanoma, head and neck squamous cell carcinoma, and colorectal cancers [8,9,10]. The immunosuppressive role of TIGIT on NK and T cells depends on its interaction with PVR. A blockade of receptor–ligand interaction by targeting TIGIT or PVR could reverse the exhaustion of NK and CD8^+^ T cells and enhance the anti-tumor immunity [3,11,12].

An important reason affecting the efficacy of immunotherapy is the infiltration of CD8^+^ T cells [13]. Although targeting various immune checkpoints alone or in combination could maximize the activation of T cells, the response to immune checkpoint blockade therapy is extremely low in the tumors with rare T cell infiltration. As the important innate immune cells and one of the most abundant cells in the tumor microenvironment, macrophages play an important role in adaptive immunity and anti-tumor response [14,15]. Macrophages could maintain the tissue homeostasis by recognizing and engulfing the foreign, damaged, or dying cells, without attacking the normal cells. The precise phagocytosis mechanism of macrophages was modulated by the balance of the “eat me” and “do not eat me” signals. Normal cells express CD47 to transmit an anti-phagocytosis signal by interacting with the signal regulatory protein alpha (SIRPα) on macrophages to avoid clearance. However, tumor cells could evade the immune surveillance via utilizing the elevated expression of CD47. A blockade of CD47/SIRPα could reactivate the phagocytosis of macrophages and enhance the anti-tumor immunity.

Simultaneously stimulating adaptive immunity and innate immunity has been proved to be a promising strategy to obtain maximum and durable anti-tumor immune response [16]. A combination of SIRPα and PD-1 blockade could result in durable anti-tumor immunity by facilitating monocyte activation, dendritic cell activation, and T cell effector functions [17]. Dual targeting CD47 and PD-L1 by fusion protein or bispecific antibody enables effective targeting to tumor cells than non-tumor cells, increased DNA sensing, DC cross-presentation, and anti-tumor T cell response [18,19]. The bispecific antibody dual targeting CD47 and TIGIT has also been designed for cancer immunotherapy (WO2020259535). However, small molecule inhibitors that dual block the CD47/ SIRPα and TIGIT/PVR pathways have not been discovered.

Repositioning existing drugs for new indications has increasingly become a smart strategy for drug discovery, with definite safety and pharmacokinetic data to potentially reduce the overall costs and shorten the development timelines [20,21]. With a systematic analysis of numerous large scale data or combined with various computational approaches, many drugs has been discovered for new applications, especially the drugs approved by the FDA [22,23]. Metformin, the first-line oral medication for type 2 diabetes (T2D), has been reported to be a classical example of drug-repositioning in cancer immunotherapy by exerting anti-inflammatory effect, and enhancing the T cell immunity through reducing the expression of the immune checkpoint PD-L1, CD73, or participating in the metabolic reprogramming, and so on [24,25,26]. An increasing number of drugs have been reported to be repositioned for cancer immunotherapy [12,27].

Here, we analyzed the expression of CD47 and PVR from the GEO database and tested the existence on the tumor cell lines by flow cytometry. Based on the structure of CD47/SIRPα and TIGIT/PVR, the small molecules from the FDA-approved drug library with dual targeting effects on SIRPα and PVR were screened by docking-based virtual screening methods. The candidate inhibitors were further verified by the blocking assays. Azelnidipine was found to simultaneously block the interaction of CD47/SIRPα and TIGIT/PVR. The binding affinity of azelnidipine to SIRPα and PVR was tested, and the binding models were analyzed. The anti-tumor effects and potential mechanism of azelnidipine were also investigated both in vitro and in vivo.

## 2. Materials and Methods

### 2.1. Gene Expression Analysis

The gene expression data of CD47 and PVR in the normal tissues and primary tumors of esophageal squamous cell carcinoma (ESCC) (GSE23400), colon tumor (GSE44076), and breast cancer (GSE42568) were downloaded from the public database National Institutes of Health Gene Expression Omnibus (GEO) (https://www.ncbi.nlm.nih.gov/geo/, accessed on 3 May 2018, 6 March 2019, and 8 June 2020, respectively). The expression level of the target proteins was analyzed with the expression matrix and platform files.

### 2.2. Virtual Screening

The crystal structures of the human SIRPα (PDB ID: 2UV3) and TIGIT/PVR complex (PDB ID: 3UDW) were acquired from the Protein Data Bank database (PDB) (https://www.rcsb.org/, accessed on 13 March 2018) [6,28]. The 3D structure of SIRPα (chain A of 2UV3) and PVR (chain C of 3UDW) was selected for docking followed by structure optimization with the software Molecular Operating Environment (MOE) (Chemical Computing Group ULC, Montreal, QC, Canada). Through the structure optimization, the target proteins were performed for protonation with the Protonate 3D function in MOE. The binding area was determined by searching the residues in the distance of 4.5Å and according to the references. The binding pocket for small molecule inhibitors was selected with the Site Finder module. A small molecule inhibitor library of FDA-approved drugs with 1686 compounds were performed the virtual screening (downloaded from the official website of Topscience (www.tsbiochem.com/), 10 May 2017). The compounds were prepared by removing extraneous salts or adjusting protonation states and minimization prior to molecular docking. Molecular docking with induced-fit parameter was conducted within MOE. The candidate compounds were screened by a comprehensive analysis of the scores and interaction details with the pocket and especially the binding area. Twenty compounds were selected for the following screening tests.

### 2.3. Cell Lines and Cell Culture

Chinese hamster ovary cell line CHOK1 stably overexpressing human or murine CD47 and TIGIT (named as CHOK1-hCD47, CHOK1-mCD47, CHOK1-hTIGIT, CHOK1-mTIGIT, respectively) were established by lenti-viral transfection. CHOK1-derived cell lines, murine melanoma cell line B16-OVA fused with EGFP (GFP^+^ B16-OVA), murine breast cancer cell line 4T1, and human colorectal cell line HT29; human esophageal squamous carcinoma cell line KYSE-70 and EC9706 were cultured in Roswell Park Memorial Institute (RPMI) 1640 medium. Human breast cancer cell line MCF-7, murine colorectal cancer cell lines CT26, MC38, and GFP^+^ MC38 cells were cultured in Dulbecco’s Modified Eagle Medium (DMEM). All the cells were cultured at 37 °C with 5% CO2, under fully humidified conditions with medium supplemented with 10% fetal bovine serum (FBS) (BI, Kibbutz Beit Haemek, Israel) and antibiotics.

### 2.4. Flow Cytometry Analysis

Cells in good growth condition were harvested and used to detect the expression of CD47 and PVR. Antibodies anti-human CD47 APC, anti-human PVR PE, anti-mouse-CD47 APC, anti-mouse-PVR APC, and matched isotype controls were purchased from eBioscience, San Diego, CA, USA. The effects of azelnidipine on the expression of CD47 and PVR was determined by incubating CT26 cells with 20 μM of azelnidipine for 24 h, or directly analyzing the tumor cells from tumor tissues of CT26-bearing mice treated with or without azelnidipine.

### 2.5. Blocking Assays

The abilities of small molecule inhibitors (SMIs) (purchased from TargetMol, Shanghai, China) to block the binding of SIRPα protein to CHOK1-CD47 cells or PVR protein to CHOK1-TIGIT cells were determined by flow cytometry, as previously reported [29]. The SMIs at indicated concentrations were incubated with Fc-tagged SIRPα or PVR protein for 30 min at 4°C, followed by incubating with the indicated cells for another 30 min. Human SIRPα protein (SIA-H5251, ACRO Biosystem, Beijing, China), mouse SIRPα protein (SIA-M5258, ACRO Biosystem, Beijing, China), human PVR protein (CD5-H82F6, ACRO Biosystem, Beijing, China), and mouse PVR protein (50259-M03H, Sino Biological, Beijing, China) were used. PE-conjugated anti-human IgG antibody was used to detect the Fc protein bind to cells. Cells incubated with the Fc proteins and anti-human IgG antibody without the SMIs serve as the positive control. The mean fluorescent intensity (MFI) of each sample was detected and recorded by a flow cytometry. The blocking efficacy of the SMIs was calculated as the equation: (MFI of the positive control-MFI of the SMI)/MFI of the positive control × 100%.

### 2.6. Microscale Thermophoresis (MST)

MST assays were performed to test the affinity of SMI-F11 (azelnidipine) to the indicated protein using the Monolith NT.115 system, as previously reported [30]. His tagged human SIRPα (SIA-H5225, ACRO Biosystem, Beijing, China), mouse SIRPα (50956-M08H, Sino Biological, Beijing, China), human PVR (CD5-H5223, ACRO Biosystem, Beijing, China), and mouse PVR (50259-M08H, Sino Biological, Beijing, China) were used. The protein was labeled with Red-NHS647 dye (NanoTemper Technologies GmhH, Munich, Germany) according to the manufacturer’s instructions. Azelnidipine was 2-fold serially diluted from 200 μM and subsequently tested. Red-NHS647 dye-labeled protein was incubated with the diluted azelnidipine, with equal volume for 5 min at room temperature; the mixture was then loaded onto standard capillaries for detection. The KD values were calculated by the analysis software (MO. Affinity Analysis, version number 2.2.4, NanoTemper Technologies GmhH, Munich, Germany).

### 2.7. Phagocytosis Assays

Bone marrow-derived macrophage (BMDM) cells were isolated from C57BL/6 or BALB/c mice and induced with 20 ng/mL of GM-CSF (Peprotech, Rocky Hill, CT, USA) for 7 days. Meanwhile, the medium was replaced with fresh medium containing the cytokine and the adherent cells were harvested. Phagocytosis assays were performed by a co-culture of the BMDM macrophages with carboxyfluorescein succinimidyl ester-labeled (CFSE^+^) or GFP^+^ tumor cells at a ratio of 1:4 in a serum-free medium at 37 °C for 4 h, in low-attachment 96-well tissue culture plates (Corning, New York, NY, USA). The cells were harvested and the macrophages were identified by a flow cytometry using anti-F4/80 antibody (eBioscience, San Diego, CA, USA). Then, 7-AAD (eBioscience) was used to exclude the dead cells. The effects of azelnidipine (20 μM) on phagocytosis by BMDM cells were tested, and the anti-CD47 antibody (clone: miap301) were used as the positive control. Phagocytosis rate was determined with the formulation: F4/80^+^GFP^+^ or F4/80^+^CFSE^+^ cells/ Total F4/80^+^ cells.

### 2.8. Tumor Models and Ex Vivo Assays

BABL/c mice were subcutaneously (*s.c.*) injected with 2 × 10^5^ CT26 cells on the right back, and C57BL/6 mice were injected with 1 × 10^6^ MC38 cells. One week later, tumor-bearing mice were randomized into indicated groups, and intraperitoneal (*i.p.*) injected with azelnidipine or normal saline every day for two weeks. Tumor volumes were measured every other day by the (a) length, (b) width, and (c) height and calculated as volume (V) = a × b × c/2. For the combinational model of azelnidipine and radiotherapy (RT), MC38 tumor-bearing mice received irradiation (IR) when the tumors reached 80–100 mm^3^. The mice received one dose of 20 Gy irradiation at the tumor local site, and followed by the treatment of azelnidipine.

Tumor-bearing mice were sacrificed at the end of the treatment. The tumors were dissected and digested into single cell with an enzyme cocktail of 100 U/mL collagenase IV; and Dnase I. The infiltration of CD8^+^ T cells at the tumor site was detected by flow cytometry by staining of anti-mouse CD45, anti-mouse CD3, anti-mouse CD8α or isotype control. The MDSCs at the tumor site were analyzed by staining of anti-mouse CD45, anti-mouse CD11b, anti-mouse Ly6C, and anti-mouse Ly6G. Spleens and draining lymph nodes were grinded and filtered into a single cell suspension. The tumor-infiltrating lymphocytes (TILs) isolated by Percoll-gradient centrifugation, splenocytes, and cells from draining lymph nodes were also performed the intracellular cytokine staining assays. The cells were stimulated with or without 20 ng/mL of phorbol 12-myristate 13-acetate (PMA, Sigma-Aldrich, St. Louis, MO, USA) and 1 µM ionomycin (Sigma-Aldrich) with the presence of protein transport inhibitor cocktail (eBioscience) for 4 h. The surface marker CD3, CD8, and the intracellular cytokine marker IFN-γ were sequentially stained according to the Foxp3/Transcription Factor Staining Buffer Set (00-5523, eBioscience). The frequency of IFN-γ secreting CD8^+^ T cells were counted according to the fluorescence minus one (FMO) control with the isotype control of IFN-γ antibody.

To establish the CD8^+^ T cell depletion model, BALB/c mice were injected with 200 μg of CD8-depleting antibody (clone: YTS169.4) or matched Rat IgG isotype control (Sigma-Aldrich) the day before tumor inoculation and as well as weekly thereafter, resulting in a total of 3 injections per mouse. Depletion efficacy of CD8^+^ T cells was verified by a flow cytometry with the blood sample of mice treated with indicated antibodies for 7 days.

### 2.9. Statistical Analysis

Statistical analysis was performed with paired or unpaired two-tailed Student’s *t*-test or two-way ANOVA with Tukey’s multiple comparisons test for analyzing differences between groups of quantitative data represented as means ± SEM, with significant differences marked on the figures. Significance levels were defined as * *p* < 0.05, ** *p* < 0.01, *** *p* < 0.001.

## 3. Results

### 3.1. CD47 and PVR Are Over-Expressed in Tumor Tissues and Cell Lines

**Figure 1:**
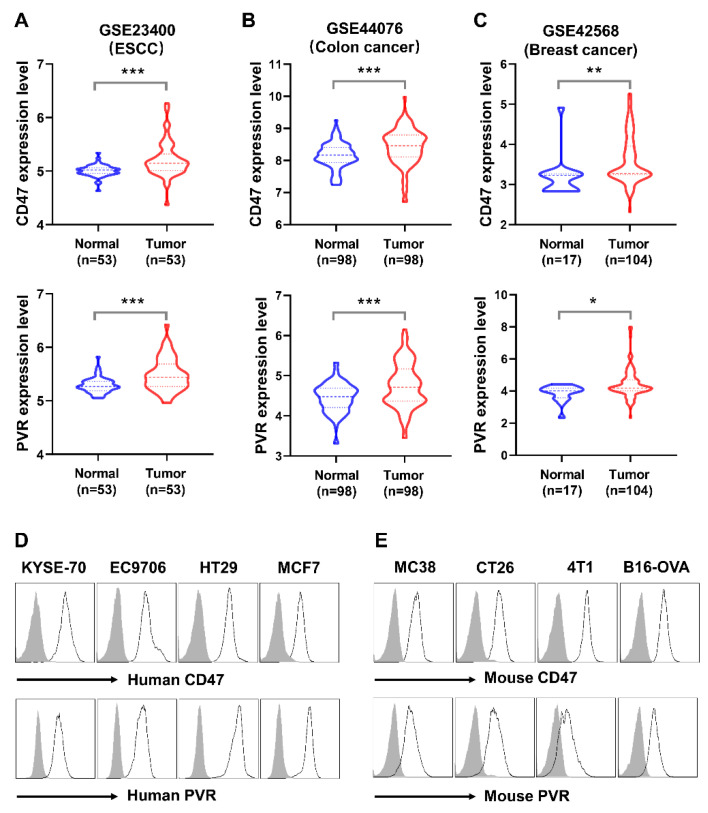
The expression of CD47 and PVR in tumor tissues and cell lines. (**A**) The expression level of CD47 and PVR in GSE23400 with the esophageal squamous cell carcinoma (ESCC) and paired normal tissues are represented. (**B**) The expression level of CD47 and PVR in GSE44076 with the colon cancer and paired normal tissues are represented. (**C**) The expression level of CD47 and PVR in GSE42568 with the breast cancer and normal tissues are represented. The sample numbers are shown in the indicated groups. The statistical analysis was conducted with paired Student’s *t*-test for the cohorts GSE23400 and GSE44076, and unpaired Student’s *t*-test for the cohort GSE42568. * *p* < 0.05, ** *p* < 0.01, *** *p* < 0.001. (**D**) Flow cytometry analysis of CD47 and PVR on human tumor cell lines. (**E**) Flow cytometry analysis of CD47 and PVR on murine tumor cell lines. The histogram lines represent indicated antibodies; shaded histogram represents the matched isotype controls.

CD47 has been reported as an important therapeutic target, and its ligation with the ligand SIRPα plays critical roles in the innate immunity [31]. The immune checkpoint TIGIT, an acknowledged exhaustion maker of both NK cell and effector CD8^+^ T cells, exerts inhibitory signals by interacting with its major ligand PVR [3]. The CD47/SIRPα and TIGIT/PVR signaling pathway jointly contribute to the immunosuppressive tumor microenvironment [32]. An increasing number of researches reported that CD47 and PVR over-expressed in various tumors. Consistent with previous reports in the public database The Cancer Genome Atlas (TCGA), CD47 and PVR expressed at high levels in Esophageal Cancer (ESCA), Colon Cancer (COAD), Head and Neck Cancer (HNSC), and Stomach Cancer (STAD) analyzed by the online tool TIMER [12,33,34,35]. Through the analysis of the public GEO database, the evaluated expression level of CD47 and PVR was also validated in esophageal squamous cell carcinoma, colon cancer, and breast cancer tissues in the GSE23400, GSE44076, and GSE42568, respectively (Figure 1A–C).

We also investigated the expression of CD47 and PVR on the tumor cell lines by flow cytometry. CD47 and PVR were highly co-expressed on the human esophageal squamous cell lines KYSE-70 and EC9706, the colorectal cancer cell line HT29, and the breast cancer cell line MCF7 (Figure 1D). The targets were also highly co-expressed in murine colorectal cancer cell lines MC38 and CT26, the metastatic breast cancer cell line 4T1, and the malignant melanoma cancer cell line B16-OVA (Figure 1E). Collectively, consistent with the previous literature, our results revealed the over-expression of CD47 and PVR in tumors, suggesting the promising role of CD47 and PVR in cancer immunotherapy.

### 3.2. Discovery of Small Molecule Inhibitors Targeting CD47/ SIRPα and TIGIT/PVR Pathways by Virtual Screening

**Figure 2:**
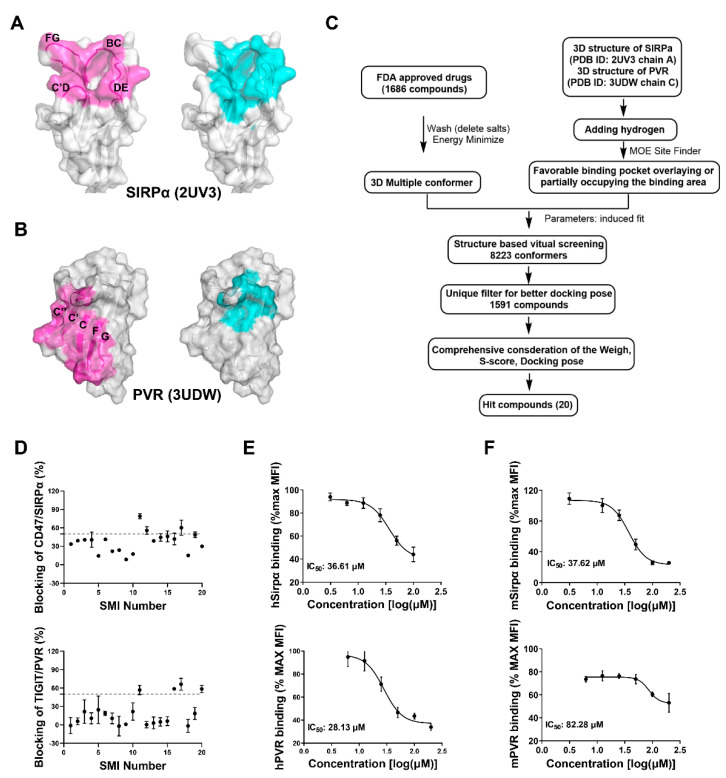
Virtual screening of small molecule inhibitors targeting SIRPα and PVR from the FDA-approved drugs. (**A**) The structure of SIRPα from PDB (PDB ID: 2UV3 chain A). The magenta represents the binding area of CD47 on SIRPα, the cyan represents the pocket for small molecule inhibitors calculated by MOE. (**B**) The structure of PVR from PDB (PDB ID: 3UDW chain C). The magenta represents the binding area of TIGIT on PVR, the cyan represents the pocket for small molecule inhibitors calculated by MOE. (**C**) The virtual screening procedures of the inhibitors. (**D**) The blocking efficacy of the small molecule inhibitors of a preliminary test at a single concentration of 100 μM. (**E**) The blocking efficacy of azelnidipine targeting human CD47/SIRPα and TIGIT/PVR. (**F**) The blocking efficacy of azelnidipine targeting mouse CD47/SIRPα and TIGIT/PVR. The data are presented as mean ± SEM and are representative of at least three independently performed experiments.

The clinical trial of the dual-target small molecules CA-170 (targeting PD-L1 and VISTA) and CA-327 (targeting PD-L1 and TIM-3) suggested the successful development of dual-target small molecule inhibitors targeting different immune checkpoints [36,37]. Dual-target small molecule inhibitor (SMI) co-targeting the CD47/SIRPα and TIGIT/PVR may also exert exciting effects in the cancer immunotherapy. The high-throughput virtual screening of small molecules with the molecular docking could facilitate the drug discovery. The high-resolution structures of CD47/SIRPα in monomer or complexes have been resolved by many groups, providing the structural basis for the virtual screening [28,38]. Considering that targeting CD47 may have relative high toxicity in the blood [39], SIRPα was selected as the target for screening. CD47 binds to SIRPα at a surface constituted by the BC, C’D, DE, and FG loops represented as magenta (Figure 2A), unlike most surface proteins that use the folding surfaces to interact with each other. The four loops form a large pocket suitable for the binding of small molecules, as the largest pocket determined by MOE (presents as cyan) overlays most of the CD47/SIRPα binding area, which was selected for the following virtual screening. As with most of the immunoglobulin superfamily surface proteins, TIGIT and PVR mutually interact through the surface formed by the β-sheets. The TIGIT binding area on PVR formed by the C, C’, C’’, F and G sheet was labeled as magenta (Figure 2B). Although the binding area is relatively smooth and not optimal for small molecule occupancy, a binding pocket close to, and partially overlapping with, the key residues on the C, C’ sheet and CC’ loop has proved to be feasible for the inhibition of protein-protein interaction (Figure 2B) [12]. The structure of the target proteins SIRPα and PVR were further processed, and the indicated pockets were set to perform the molecular docking. The protein and the small molecules from the FDA-approved drug library were prepared and the small molecules were screened according to the procedures illustrated in Figure 2C. Finally, 20 candidate small molecules were obtained with the comprehensive consideration of the docking parameters (Supplemental Table S1).

### 3.3. Screening and Validation of the Candidate Small Molecule Inhibitors by Blocking Assay

The small molecule inhibitor could not only bind to the target; most importantly, it should interfere with the receptor–ligand interactions. Subsequently, the candidate inhibitors were screened by the blocking assays with the established CD47 and TIGIT over-expressing cell lines. The test was initialed with a preliminary screening of the candidates with a single concentration (Figure 2D). Generally, the SMI blocking efficacy to the CD47/SIRPα interaction was much higher than that of the TIGIT/PVR. Small molecule inhibitors SMI-F11, SMI-F12, SMI-F17, and SMI-F19 could block the interaction of CD47 and SIRPα with more than 50% blocking efficacy, while SMI-F11, SMI-F16, SMI-F17, and SMI-F20 show better activities in interfering with the TIGIT/PVR interaction. Therefore, the SMI-F11 and SMI-F17 may exert dual blocking effects in the CD47/SIRPα and TIGIT/PVR pathways. Blocking assays with a concentration gradient of the SMI were further performed to validate the preliminary screening. SMI-11 (azelnidipine) could efficiently block both the human CD47/SIRPα and TIGIT/PVR interactions with the IC_50_ of about 36 μM and 28 μM, respectively (Figure 2E), while SMI-17 could not exert blocking effects in a concentration-dependent manner. The similar blocking assays with the mouse CD47 and TIGIT over-expressing cells were also performed. Azelnidipine could also block both the mouse CD47/SIRPα and TIGIT/PVR interactions with the IC_50_ of about 37 μM and 82 μM, respectively (Figure 2F). Considering that most pairs of the immune checkpoints interact with the corresponding ligand in parallel forms, the blocking assays with the PD-1/PD-L1 interaction system was also conducted to test the specificity. Azelnidipine could not block the interaction of human PD-1/PD-L1, suggesting that azelnidipine has specificity by blocking the CD47/SIRPα and TIGIT/PVR interactions (Supplemental Figure S1).

### 3.4. The Binding Activity and Model of Azelnidipine to SIRPα and PVR

**Figure 3:**
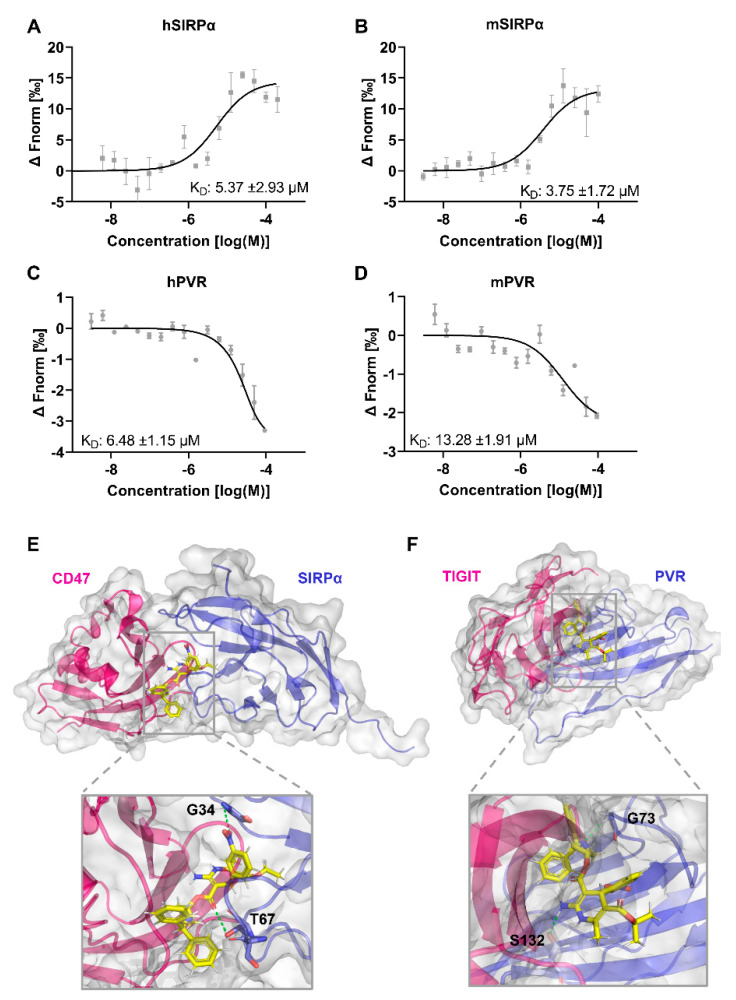
The binding affinity and model of azelnidipine to SIRPα and PVR. Binding of azelnidipine to human and mouse SIRPα or PVR was examined by the MST. (**A**,**B**) Dose response curves of azelnidipine binding to human SIRPα (**A**) and PVR (**B**). (**C**,**D**) Dose response curves of azelnidipine binding to mouse SIRPα (**C**) and PVR (**D**). The K_D_ values were calculated with analysis software (MO. Affinity Analysis v2.2.4). Data are representative of at least three independent experiments. (**E**) The binding model and detail of azelnidipine to SIRPα. The protein surface of CD47 (magenta) and SIRPα (blue) and azelnidipine (yellow) are shown, with the interactions labeled. (**F**) The binding model and detail of azelnidipine to PVR. The protein surfaces of TIGIT (magenta) and PVR (blue) and azelnidipine (yellow) are shown, with the interactions labeled.

After determining that azelnidipine could dually block both the CD47/SIRPα and TIGIT/PVR pathways, its affinity to the targets SIRPα and PVR was further determined using the MST method. Azelnidipine could bind to human SIRPα and PVR with the comparable K_D_ values of 5.37 μM and 6.48 μM (Figure 3A,B). Considering the high sequence identity and similar structure of the human and mouse SIRPα or PVR, MST assays was also conducted to test the binding of azelnidipine to the mouse proteins. Azelnidipine could bind the mouse SIRPα with a K_D_ value of 3.75 μM comparable to human SIRPα, while it binds to mouse PVR with a K_D_ value of 13.28 μM (Figure 3C,D). The binding of azelnidipine to mouse PVR is slightly weaker, and this may explain the difference of the blocking efficacy between the human and mouse TIGIT/PVR.

To elucidate the possible mechanism of the dual targeting of azelnidipine to SIRPα and PVR, the binding models were analyzed. The convoluted interacting face of CD47 with several β-sheets and loops occupy the surface of SIRPα comprised of the BC, C’D, DE, and FG loops. With analysis of the docking results, azelnidipine interacts with the residues G34 on the BC loop and T67 on the DE loop of SIRPα, among which T67 was critical for receptor–ligand ligation by interacting with CD47 through the residues N27 on the BC loop and R103 on the G β-sheet (Figure 3E). By interacting with SIRPα across BC and DE loops, azelnidipine could partially occupy the binding area on SIRPα and interfere the CD47/SIRPα ligation. Azelnidipine could also nicely target the TIGIT binding area on PVR and form a barrier for the protein-protein interaction. In detail, azelnidipine interacts with the residues G73 on the CC’ loop and S132 on the G β-sheet (Figure 3F). Although TIGIT binds to PVR without interacting with G73, the S72 and S74 are involved in the formation of the key residue pairs, and the interaction between azelnidipine and G73 forms steric hindrance for TIGIT/PVR ligation. The residue S132 on PVR interacts with the N58 on TIGIT, and functions as a critical residue for TIGIT/PVR interaction [40].

### 3.5. Azelnidipine Could Enhance the Macrophages-Mediated Phagocytosis of Tumor Cells In Vitro

**Figure 4:**
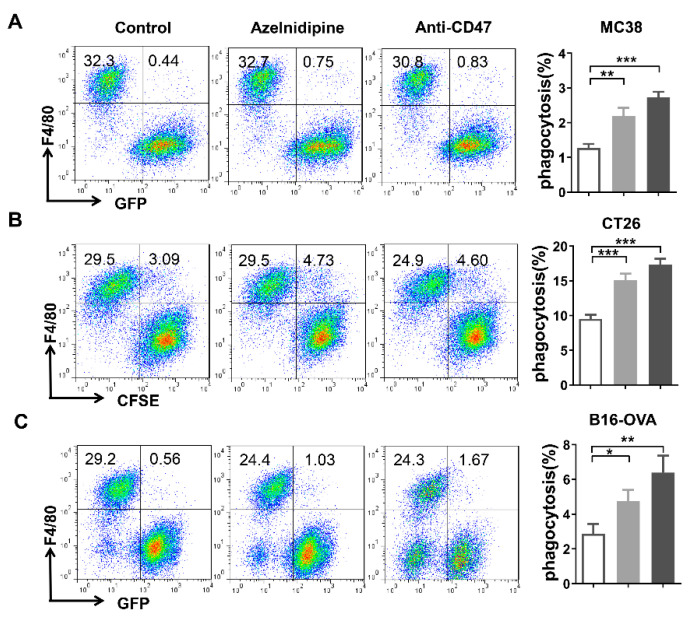
Azelnidipine enhances macrophage mediated phagocytosis of the tumor cells. The effects of the azelnidipine on the phagocytosis of the macrophages were tested by a co-culture of the tumor cells and BMDM cell derived from C57BL/6 or BALB/c mice. GFP^+^ MC38 cells (**A**), CFSE-labeled CT26 cells (**B**), and GFP^+^ B16-OVA (**C**) were co-cultured with the BMDM cells at the ratio of 4:1 in serum-free medium at 37℃ for 4 h with or without the presence of 20 μM of azelnidipine or the positive control anti-CD47 antibody. GFP^+^ or CFSE^+^ BMDMs were detected by a flow cytometry. Representative flow cytometry plots and the summary data of at least three independent experiments were shown. *, *p* < 0.05, **, *p* < 0.01, ***, *p* < 0.001, Student’s *t*-test.

CD47 mediates tumor evasion by serving as a “do not eat me” marker and transmitting the anti-phagocytic signal to macrophages. Macrophages-mediated phagocytosis was one of the major mechanisms of CD47 targeted therapies. Here, the effects of azelnidipine on the macrophage phagocytosis of tumor cells were explored by a co-culture of the BMDM cells and various types of tumor cells. As a result, a blockade of CD47/SIRPα interaction by azelnidipine could significantly enhance the phagocytosis of the MC38, CT26, and B16-OVA cells by BMDM cells slightly inferior to the anti-CD47 positive control (Figure 4).

### 3.6. Azelnidipine Could Inhibit MC38 Tumor Growth Combined with Radiotherapy

**Figure 5:**
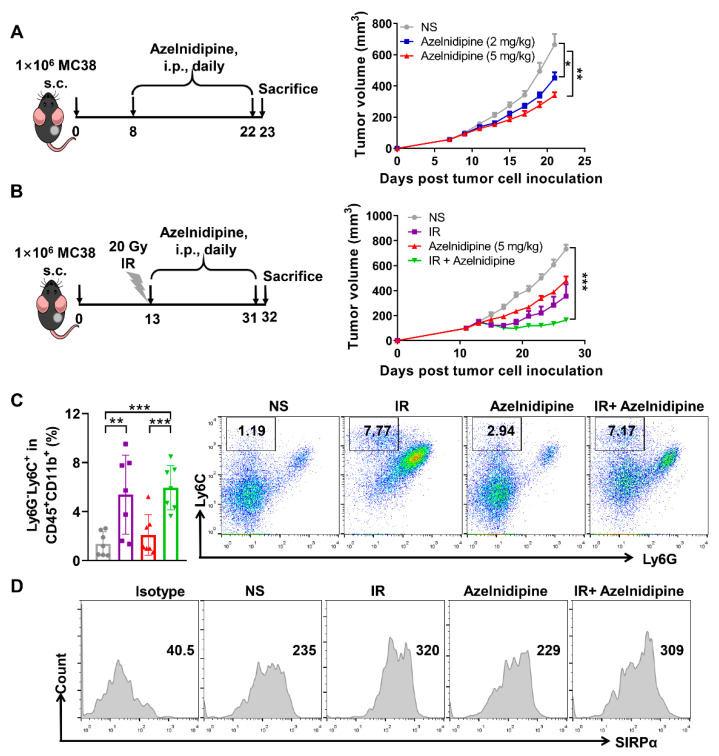
The antitumor effects of azelnidipine alone or combined with radiotherapy in MC38 tumor model. Azelnidipine alone or combined with radiotherapy significantly inhibit MC38 tumor growth. (**A**) Mice were *i.p.* injected with 2 or 5 mg/kg of azelnidipine each day for 2 weeks as in the schematic diagram. Tumor growth curve of MC38 tumor-bearing mice treated with normal saline or azelnidipine alone (*n* = 9 or 10). (**B**) Mice were *i.p.* injected with 5 mg/kg of azelnidipine each day followed by 20 Gy of local IR as in the schematic diagram. Tumor growth curve of MC38 tumor-bearing mice treated with normal saline, IR, azelnidipine alone or combined (*n* = 7). (**C**) Representative flow cytometry plots and summary data of the MDSCs in the tumor tissues of each group (*n* = 7). (**D**) Representative flow cytometry histograms of the expression of SIRPα on MDSCs in the tumor tissues of each group (*n* = 7). *, *p* < 0.05, **, *p* < 0.01, ***, *p* < 0.001. Two-way ANOVA with Tukey’s multiple-comparisons test (**A**,**B**) and Student’s *t*-test (**c**) were conducted.

The effects of a CD47/SIRPα blockade by azelnidipine were further explored in the MC38 tumor model, which has a large quantity of intratumoral macrophages and was widely used for the CD47 targeted anti-tumor study [18]. Tumor-bearing mice with visible tumors of about 100 mm^3^ were intraperitoneally treated with 2 and 5 mg/kg of azelnidipine. Both dosages of azelnidipine could significantly restrict the growth of MC38 tumors (Figure 5A). At the clinic, radiotherapy is frequently adopted for the tumor eradication. Moreover, the tumor local irradiation (IR) could augment the infiltration of monocytic myeloid-derived suppressor cells (M-MDSCs) (CD11b^+^ Ly6C^hi^Ly6G^-^) in tumor tissues [41]. The M-MDSCs could function themselves or differentiated into macrophages to phagocytize tumor cells [42,43]. We therefore hypothesized that pretreatment of local IR might synergize with azelnidipine to further enhance tumor control. Tumor-bearing mice received locally IR at 20 Gy when the tumors reached approximate 100 mm^3^, and followed by daily treatment of azelnidipine. Consistent with our inference, a combination of azelnidipine and IR could remarkably suppress tumor growth (Figure 5B). Mechanistically, the analysis of the tumor infiltrating immune cells confirmed that local IR of MC38 tumors indeed significantly augment the frequency of Ly6G^-^Ly6C^+^ M-MDSCs, with or without the combination of azelnidipine (Figure 5C). The expression level of SIRPα on the intratumoral M-MDSCs of tumor-bearing mice received local IR also significantly increased (Figure 5D). Irradiation damages the tumor tissues and increases infiltration and SIRPα expression on MDSCs, and combined with azelnidipine that blocks CD47/SIRPα interaction, it can enhance MDSC cell-mediated phagocytosis of tumor cells, thereby remarkably inhibiting the tumor growth.

### 3.7. Azelnidipine Could Significantly Inhibit the Tumor Growth and Elicit Anti-Tumor T Cell Immune Response

**Figure 6:**
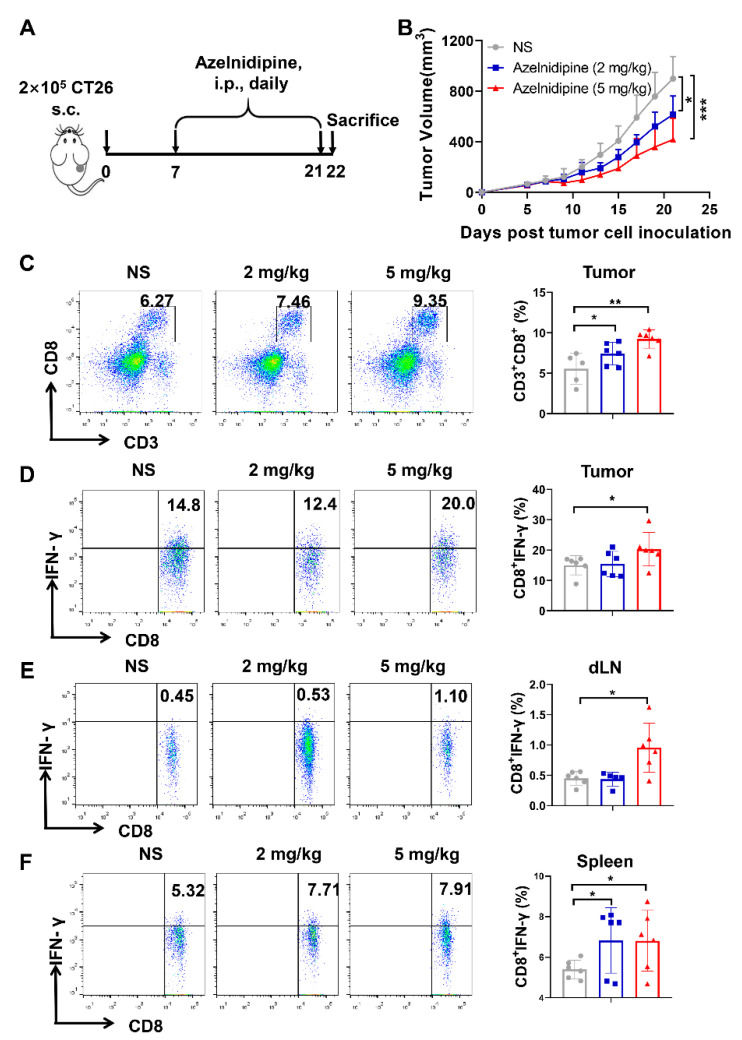
The antitumor effects of azelnidipine in CT26 tumor model. (**A**) CT26 tumor-bearing mice were *i.p.* administrated with 2 or 5 mg/kg of azelnidipine each day for 2 weeks as in the schematic diagram. (**B**) Tumor growth curve of CT26 tumor-bearing mice treated with normal saline or azelnidipine (*n* = 7). *, *p* < 0.05, ***, *p* < 0.001.Two-way ANOVA with Tukey’s multiple-comparisons test were conducted. (**C**) Representative flow cytometry plots and summary data of the frequency of CD8^+^ T cells in the tumor tissues. Representative intracellular IFN-γ staining plots and summary data of the tumor infiltrating CD8^+^ T cells (**D**), tumor-draining lymph node cells (**E**) and splenocytes (**F**). The frequency of IFN-γ secreting CD8^+^ T cells were counted according to the fluorescence minus one (FMO) control with the isotype control of IFN-γ antibody. *, *p* < 0.05, **, *p* < 0.01, Student’s *t*-test were conducted.

It is reported that a CD47/SIRPα blockade and TIGIT/PVR blockade could elicit significant anti-tumor responses [11,44]. We further investigated the anti-tumor effects of azelnidipine in a CT26 model, which is widely used for CD47/SIRPα and TIGIT/PVR blockade therapy and the exploration of the T cell immune response. By targeting PVR, azelnidipine could significantly restrict the CT26 tumor growth at both administration dosages (Figure 6A,B). Azelnidipine treatment could also remarkably increase the frequency of CD8^+^ T cells in the tumor tissues (Figure 6C). More importantly, except the increase in cell number, the IFN-γ secretion function of the tumor infiltrating CD8^+^ T cells also enhanced (Figure 6D). CD8^+^ T cells are the main effector anti-tumor cells; thus, the augment quantity and enhanced function of the CD8^+^ T cells might jointly lead to the tumor reduction. The systemic immune response was also explored; a low dosage of azelnidipine could enhance the secretion of CD8^+^ T cells in the spleen, while a high dosage of azelnidipine could significantly boost the secretion of IFN-γ in both the spleen and the tumor draining lymph node (Figure 6E,F). Collectively, the above results proved that targeting PVR with azelnidipine could significantly inhibit the tumor growth and elicit anti-tumor T cell immune response.

Since the function of CD8^+^ T cells significantly enhanced with the azelnidipine treatment, we further explored whether the anti-tumor effects of azelnidipine depends on the CD8^+^ T cells. A CD8^+^ T cells depletion CT26 tumor model was well established (supplemental Figure S2a,b). In the Rat IgG treated groups, azelnidipine could significantly inhibit tumor growth as in Figure 6B, while the anti-tumor effect was abrogated with CD8^+^ T cells depleted (supplemental Figure S2c). These results suggested that the anti-tumor effects of azelnidipine was CD8^+^ T cell dependent, as with most immune checkpoint blockers.

Furthermore, we also analyzed the infiltration of MDSCs and the expression of SIRPα with the CT26 tumor-bearing mice, same as in the MC38 tumor model in Figure 5. Consistent with Figure 5, there are large quantity of MDSCs with a high expression level of SIRPα in the tumor tissues (supplemental Figure S2d,e). Although the infiltration of MDSCs and their expression level of SIRPα show no significant difference in the NS and azelnidipine groups, the inhibitory pathway of CD47/SIRPα exists in the CT26 tumor model, which provides the rationality for a CD47/SIRPα blockade. We also explored the effects of azelnidipine on the expression of CD47 and PVR. In vitro, azelnidipine could slightly inhibit the expression of PVR on CT26 cells without suppressing the expression of CD47 analyzed by a flow cytometry (supplemental Figure S3a,b). Meanwhile, we also analyzed the expression of CD47 and PVR on CT26 tumor cells derived from the tumor tissues. Consistent with the results in vitro, azelnidipine could inhibit the expression of PVR but not that of CD47 (Supplemental Figure S3c,d). Thus, azelnidipine could not only block the TIGIT/PVR pathway, but also reduce the expression of PVR.

## 4. Discussion

CD8^+^ T cells are important effector cells in anti-tumor immunity, while innate immunity is indispensable to initiate adaptive immunity and recruit activated T cells to reach the action site. The success of bispecific antibody targeting of both innate and adaptive immune checkpoints has firmly established the proof of the concept that combinational therapy of the innate and adaptive immunity has a broad application prospect [17]. A CD47/SIRPα blockade not only re-motivates the innate immunity by enhancing the macrophages phagocytosis, but also enhances the adaptive immunity by cross-priming T cells by DC cells [44]. A TIGIT/PVR blockade could reverse the exhaustion of both T cells and NK cells, resulting in significant and durable anti-tumor effects [3]. Combinational therapy targeting CD47/SIRPα and TIGIT/PVR pathways is of important clinical significance.

Although CD47 was upregulated on tumor cells, its ubiquitous expression on the normal cell, especially the hematopoietic cells, has been a major concern for CD47-targeted therapy. The side effects of hematological toxicity caused by non-specific clearance with the anti-phagocytic signal released may severely restrict the application of a CD47 blockade. With relatively restricted expression, an SIRPα blockade could enhance the macrophage mediated innate immunity with reduced side effects such as anemia. Therapeutics targeting SIRPα including antibodies and macrocyclic peptide are rapidly developing [45]. PVR, the major ligand of TIGIT, plays a pivotal role in multitudinous biological processes, especially the tumor immune escape. From the resolved structure, PVR shows advantages for drug screening with a relative rough surface compared with TIGIT [12]. On the other hand, as a shared ligand, a PVR blockade is expected to interfere in both TIGIT/PVR and CD96/PVR negative signal pathways [46]. Collectively, SIRPα and PVR was selected as the targets.

Drug repositioning plays an increasingly important role in the field of cancer immunotherapy. Numerous methods or tools are developed to accelerate the identification of new drug-target interactions or new target-disease relations, among which computational molecular docking based on the structures to predict binding site complementarity between the ligand and target is a promising approach [22]. Resolution of the crystal structure of the immune checkpoints makes it feasible to acquire small molecule inhibitors by docking-based virtual screening [6,38]. In this study, compounds approved by the FDA were screened by docking to the potential binding pockets of SIRPα and PVR identified with the structural analysis of SIRPα and PVR. Excitingly, the FDA-approved drug azelnidipine could occupy the binding areas of CD47/SIRPα and TIGIT/PVR and interact with the key residues.

The mainstream strategy for an immune checkpoint blockade is the specific antibodies. Low molecular weight inhibitors, including high-affinity protein mutants, peptides, and small molecules, have also been designed for cancer treatment [37]. Large numbers of small molecule inhibitors targeting PD-1/PD-L1 have been developed. Using virtual screening in combination with comprehensive methods, we redefined the function of the small molecule compound liothyronine in blocking the interaction between TIGIT and PVR for cancer immunotherapy [12]. Furthermore, a single drug with function in modulating the activity of multiple targets over single-targeted or combination therapy has potential advantages. Multiple target molecules with dual activity shows a more predictable pharmacokinetic profile and less toxicity than a cocktail of multiple molecules [47]. Small molecule CA-170 for a dual blockade of PD-L1 and VISTA, and CA-327 for a dual blockade of PD-L1 and TIM-3 have been proceeded to clinical trials [36]. Here, we firstly demonstrated that azelnidipine dually targeting SIRPα and PVR could be a promising anti-tumor modality in cancer immunotherapy. Although the effect of azelnidipine for dual blocking of CD47/SIRPα and TIGIT/PVR interactions indeed occurs within one single model, it is hard to distinguish the contribution to the anti-tumor effects of either single blockade. In addition, since there are currently no specific small molecule inhibitors for TIGIT/PVR and CD47/SIRPα, it is not possible to compare the effect of a single blockade of azelnidipine for the time being.

Calcium channel blockers are widely used for the treatment of hypertension as a first-line drug and have been reported to have immunosuppressive roles in inhibiting the functions of T cells and macrophages [48,49,50]. Other researches demonstrated that different calcium channel blockers have different regulatory effects on the cytokine secretion of peripheral blood mononuclear cells [51]. The research about the immune-regulatory role of azelnidipine is limited and controversial. On the one hand, azelnidipine was reported to inhibit the differentiation and activation of THP-1 macrophages and the cytokine secretion of peripheral blood mononuclear cells [52,53]. It has also been reported that azelnidipine can enhance the secretion of IL-12 p40 mediated by immune complex, without facilitating the differentiation of Th17 cells, which induces autoimmunity or impair anti-tumor immunity [54]. Despite the dispute and requirement of further exploration, azelnidipine has been reported to possess anti-tumor effects [55,56]. Azelnidipine can inhibit the growth of various tumor cells in vitro, and significantly inhibit the tumor growth in vivo, and the underlying mechanism remains unclear. In this study, we demonstrated that azelnidipine could dual target SIRPα and PVR, simultaneously block the negative signal pathway, enhance the phagocytosis of tumor cells by macrophages, and significantly inhibit tumor growth. Moreover, azelnidipine could not only block the TIGIT/PVR pathway, but also reduce the expression of PVR, which may further enhance the anti-tumor effects. Our research may provide a potential mechanism for the tumor treatment by azelnidipine.

## 5. Conclusions

Here, we examined the over-expression of CD47 and PVR in a broad spectrum of cancers and tumor cell lines. We firstly discovered that an FDA-approved anti-hypotensive drug azelnidipine could be repositioned for cancer immunotherapy by dual targeting SIRPα and PVR through docking-based virtual screening. Azelnidipine could simultaneously bind to the key residues of SIRPα and PVR and block the interaction of both CD47/SIRPα and TIGIT/PVR. Azelnidipine could remarkably enhance the engulfment of tumor cells by macrophages in vitro. Irradiation could enhance the infiltration of MDSCs and upregulation of its expression of SIRPα, and azelnidipine alone or combined with irradiation could significantly inhibit the growth of MC38 tumors. Additionally, azelnidipine exerted anti-tumor effects by enhancing the quantity and activation of CD8^+^ T cells in the local tumor site, draining the lymph node and spleen. Our research provides a promising candidate for cancer immunotherapy, bridging the innate and adaptive immunity by a CD47/SIRPα and TIGIT/PVR blockade.

## Data Availability

The datasets generated during and/or analyzed during the current study are available from the corresponding author on reasonable request.

## Supplementary Materials

The following are available online at https://www.mdpi.com/article/10.3390/biom11050706/s1, Figure S1: The blocking efficacy of PD-1/PD-L1 interaction by azelnidipine. Figure S2: The antitumor effects of azelnidipine in CD8^+^ T cell depleted CT26 tumor model. Figure S3. The effects of azelnidipine on the expression of CD47 and PVR on CT26 tumor cells. Table S1: Candidate compounds through virtual screening.
