# Supplementary material for: Repositioning Azelnidipine as a Dual Inhibitor Targeting CD47/SIRPα and TIGIT/PVR Pathways for Cancer Immuno-Therapy
Source: Biomolecules. 2021 May 10;11(5):706. doi: 10.3390/biom11050706 (PMC8150775; doi:10.3390/biom11050706)
Supplement: Supplementary file 1 [file biomolecules-11-00706-s001.zip › biomolecules-1163324-supplementary.pdf]

**Supplemental Materials.**

There are three supplemental Figures and one supplemental Table.

**Supplemental figure and legend**

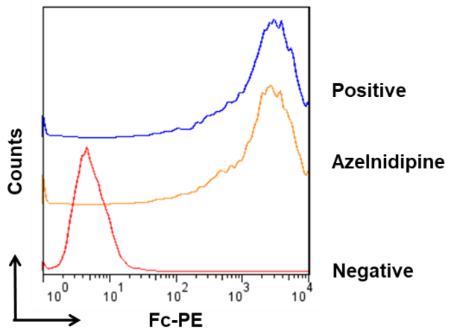


**Figure S1. The blocking efficacy of PD-1/PD-L1 by Azelnidipine.** (A) The representative FACS histogram of blocking assay of Azelnidipine (100 μM) with CHOK1-hPD-L1 cells and PD-1-Fc protein (blue histogram). PD-1-Fc protein was mixed with or without Azelnidipine for 30 min, and then incubate with CHOK1-hPD-L1 cells for another 30 min, followed by staining of anti-Fc-PE antibody. Cells stained with PD-1-Fc protein coupled with anti-Fc-PE antibody served as the positive control, and only with anti-Fc-PE antibody served as the negative control.

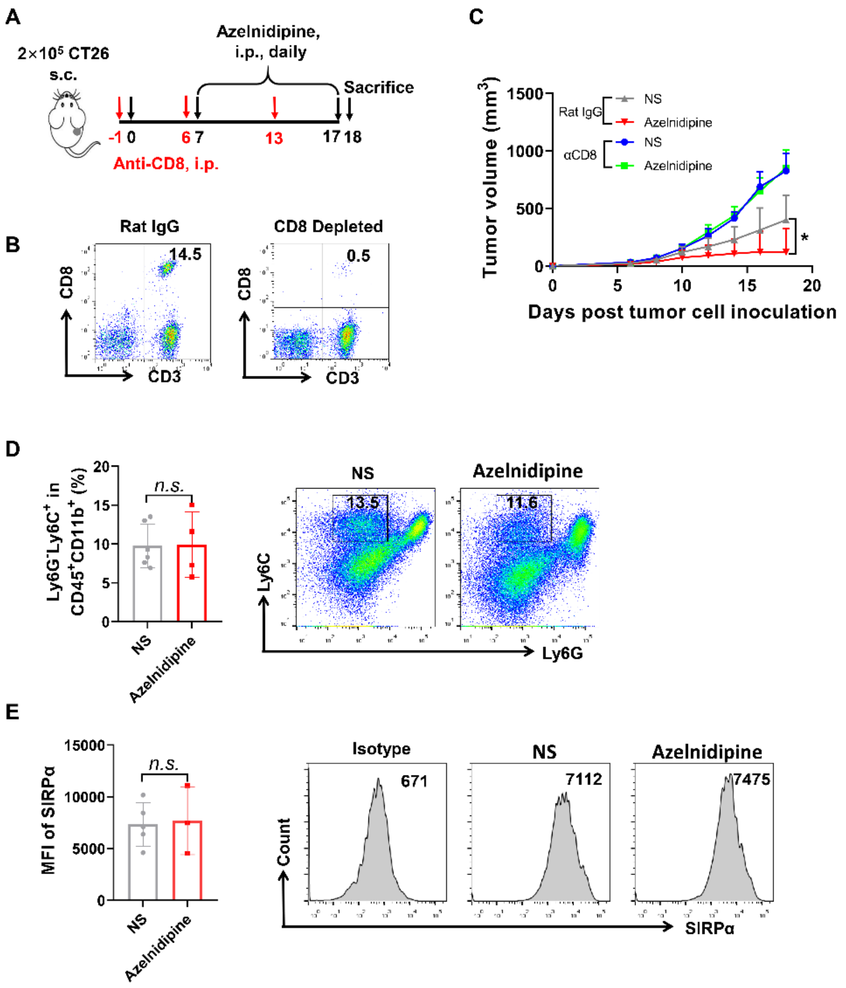


**Figure S2. The antitumor effects of Azelnidipine in CD8$^{+}$ T cell depleted CT26 tumor model.** (A) CT26 tumor-bearing mice were *i.p.* administrated with 5 mg/kg of Azelnidipine daily as in the schematic diagram. (B) Blood samples of the mice treated with 200 μg anti-CD8 (YTS 169.4) or Rat IgG control for seven days were used to analyze the depletion efficacy by flow cytometry. (C) Tumor growth curve of CT26 tumor-bearing mice treated with normal saline or Azelnidipine alone ($n$ = 5 or 6). (D) Representative flow cytometry plots and summary data of the MDSCs in the tumor tissues of the Rat IgG treated groups (NS and Azelnidipine). (E) Representative flow cytometry histograms and summary data of the expression of SIRPα on M-MDSCs in the tumor tissues of the Rat IgG treated groups (NS and Azelnidipine). *, $P$<0.05.

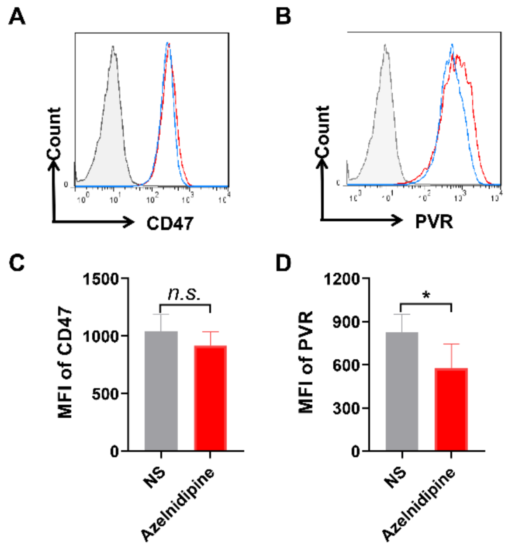


**Figure S3. The effects of Azelnidipine on the expression of CD47 and PVR on CT26 tumor cells.** (A and B) CT26 cells were incubated with 20 μM Azelnidipine or vehicle control for 24h, and analyzed the expression of CD47 (A) and PVR (B). The gray shaded histogram represents the isotype, the red line represents the cells treated with vehicle control, and the blue line represents the cells treated with Azelnidipine. (C and D) Tumor cells ($CD45^-$) derived from the tumor tissues of CT26 tumor-bearing mice treated with normal saline (NS) or 5 mg/kg Azelnidipine for twelve days, were analyzed for the expression of CD47 (C) and PVR (D). *n.s.* means not significant. *, $P<0.05$.

Table.S1 Candidate compounds through virtual screening targeting SIRPα and PVR.

| | | |
|---|---|---|
| 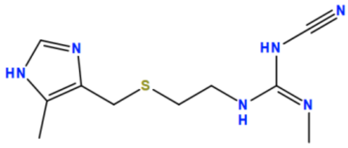 | 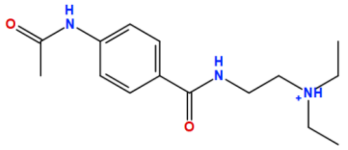 | 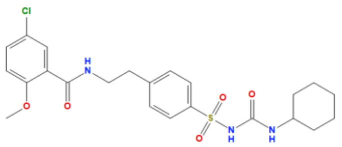 |
| F1: Cimetidine | F2:Acecainide | F3: Glyburide |
| 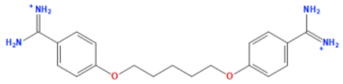 | 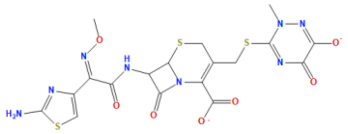 | 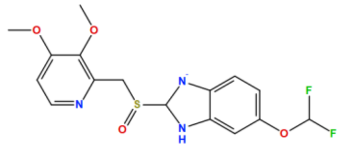 |
| F4: Pentamidine isethionate | F5: Ceftriaxone sodium | F6: Pantoprazole sodium |
| 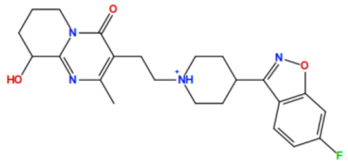 | 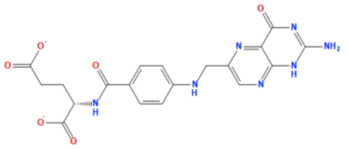 | 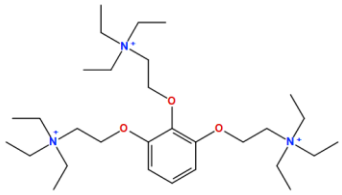 |
| F7: Paliperidone | F8: Folic acid | F9: Gallamine triethiodide |
| 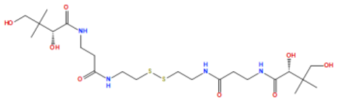 | 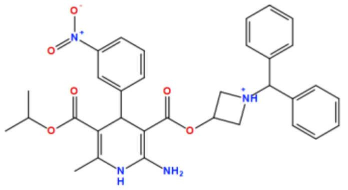 | 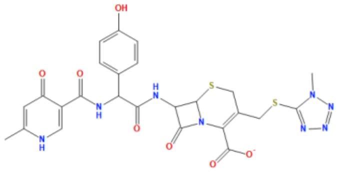 |
| F10: Pantethine | F11: Azelnidipine | F12: Cefpiramide acid |
| 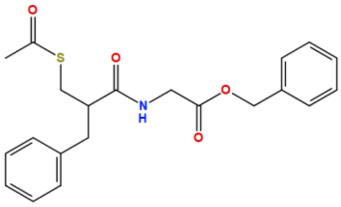 | 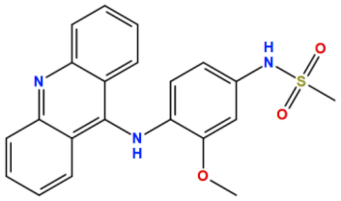 | 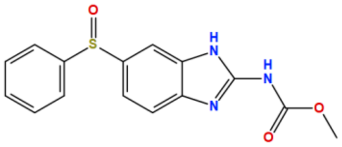 |
| F13: Racecadotril | F14: Amsacrine | F15: Oxfendazole |
| 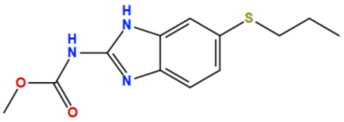 | 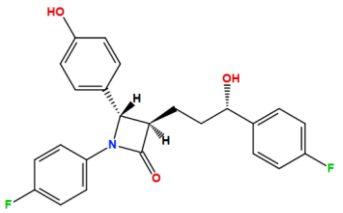 | 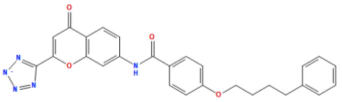 |
| F16: Albendazole | F17: Ezetimibe | F18: Pranlukast |
| 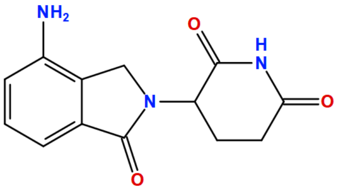 | 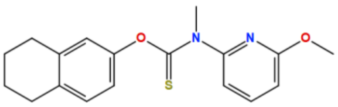 | |
| F19: Lenalidomide | F20: Liranaftate | |
